# Occupational Moral Injury and Professional Quality of Life Among a Group of Greek Nurses: A Cross-Sectional Study

**DOI:** 10.3390/healthcare14131969

**Published:** 2026-07-02

**Authors:** Evangelos C. Fradelos, Anna Patsopoulou, Maria Robie, Eirini Stylianou, Aikaterini Toska, Pavlos Sarafis, Ioanna V. Papathanasiou, Anna Mauroforou, Maria Saridi

**Affiliations:** 1 Department of Nursing, University of Thessaly, 41500 Larissa, Greece; pats.anna@yahoo.com (A.P.); ktoska@uth.gr (A.T.); psarafis@uth.gr (P.S.); iopapathanasiou@uth.gr (I.V.P.); amavroforou@uth.gr (A.M.); msaridi@uth.gr (M.S.); 2 Faculty of Medicine, University of Thessaly, 41500 Larissa, Greece; mariarombie@gmail.com (M.R.); stylianoueirini@yahoo.gr (E.S.)

**Keywords:** occupational moral injury, professional quality of life, compassion satisfaction, burnout, secondary traumatic stress, nurses

## Abstract

**Background**: Occupational moral injury is associated with nurses’ psychological well-being, yet evidence linking it to professional quality of life remains limited, and no validated Greek version of the Occupational Moral Injury Scale (OMIS) has been available. **Methods**: A cross-sectional study was conducted among 301 nurses from two public hospitals in Greece. Using convenience sampling, 350 questionnaires were distributed, yielding an 86.0% response rate. Participants completed the Occupational Moral Injury Scale (OMIS) and the Professional Quality of Life Scale Version 5 (ProQOL-V). The OMIS was translated and culturally adapted into Greek through a forward–backward translation process, and its validity and reliability were assessed using confirmatory factor analysis and internal consistency testing. **Results**: The Greek OMIS demonstrated excellent internal consistency (Cronbach’s α = 0.95). CFA provided preliminary support for the proposed factorial structure, although model fit indices indicated a marginal-to-moderate fit (CFI = 0.887, TLI = 0.866, SRMR = 0.070 and GFI 0.902). Participants reported relatively high compassion satisfaction (M = 37.30, SD = 7.35) and moderate levels of burnout (M = 23.36, SD = 5.06) and secondary traumatic stress (M = 22.13, SD = 7.28). Betrayal was negatively associated with compassion satisfaction (r = −0.151, *p* = 0.009) and positively associated with burnout (r = 0.427, *p* < 0.001) and secondary traumatic stress (r = 0.366, *p* < 0.001). Regression analyses showed that dimensions of moral injury were associated with burnout, secondary traumatic stress, and compassion satisfaction, with betrayal showing the strongest associations. **Conclusions**: Occupational moral injury was associated with poorer professional quality of life, underscoring the need for organizational support, ethical resources, and clinical supervision to promote healthcare professionals’ well-being and resilience.

## 1. Introduction

Occupational quality of life refers to the extent to which an individual’s professional role influences overall well-being, either positively or negatively, and is not limited to healthcare professionals [1]. Compassion is central to healthcare professionals’ identity and is embedded in ethical frameworks such as the Hippocratic Oath. However, sustained exposure to patient suffering and clinical complexity may impose a substantial psychological burden.

Prolonged exposure to physical, psychological, and emotional stressors can contribute to burnout and secondary traumatic stress [2,3]. Related constructs such as compassion fatigue, post-traumatic stress, and compassion satisfaction are widely discussed in healthcare literature, although they are not exclusive to nursing practice. The concept of professional quality of life originated in social work and is rooted in trauma-related research on helping professions [1,4,5].

Although conceptually related, moral injury, burnout, and compassion fatigue are distinct constructs. Burnout reflects chronic occupational stress characterized by emotional exhaustion, depersonalization, and reduced personal accomplishment. Compassion fatigue refers to the emotional strain associated with repeated exposure to suffering [1]. In contrast, moral injury arises when individuals perpetrate, fail to prevent, witness, or are subjected to events that violate deeply held moral or ethical beliefs. While these conditions may coexist, moral injury is fundamentally grounded in ethical conflict rather than stress alone [6].

Moral injury has been defined as the psychological distress resulting from actions, or the failure to act, that violate an individual’s moral or ethical values [6]. Such experiences may involve personal actions, witnessing others’ actions, or perceived betrayal by authority figures [7]. Initially conceptualized in military populations, moral injury has increasingly been examined in healthcare settings, where professionals may encounter ethically challenging situations that conflict with their moral values [8,9].

Healthcare environments are characterized by high clinical demands, ethical dilemmas, and resource constraints, all of which may adversely affect healthcare professionals’ psychological well-being [8,10]. Individuals experiencing moral injury may report increased psychological distress, reduced well-being, and impaired professional functioning.

The COVID-19 pandemic further intensified these challenges, exposing healthcare professionals to resource scarcity, high mortality rates, and ethically complex decision-making under extreme pressure [11,12,13,14]. Additional stressors such as extended working hours, social isolation, and increased infection risk further exacerbated psychological strain [15,16].

Moral injury has been associated with a range of adverse mental health outcomes, including burnout, anxiety, depression, and suicidal ideation [17,18]. Reported prevalence among healthcare professionals ranges from 34.2% to 50% [19,20,21,22]. Despite increasing international evidence, research on occupational moral injury in Greece remains limited. This is particularly important given systemic pressures within the Greek healthcare system, including workforce shortages, resource limitations, and the long-term effects of economic austerity and the COVID-19 pandemic. These factors may increase exposure to morally challenging situations and heighten vulnerability to moral injury. Moreover, a validated Greek version of the Occupational Moral Injury Scale (OMIS) has not previously been available, limiting systematic assessment in this context.

This study aimed to examine the association between occupational moral injury and professional quality of life among healthcare professionals working in the Greek public healthcare sector. A secondary aim was to translate, culturally adapt, and evaluate the psychometric properties of the Greek version of the Occupational Moral Injury Scale (OMIS). Specifically, the study investigated the relationships between dimensions of moral injury (betrayal, commission with agency, commission under duress, omission, and witnessing) and professional quality-of-life outcomes, including compassion satisfaction, burnout, and secondary traumatic stress.

We hypothesize that:

**H1.**  *Higher moral injury will be associated with higher burnout.*

**H2.**  *Higher moral injury will be associated with higher secondary traumatic stress.*

**H3.**  *Higher moral injury will be associated with lower compassion satisfaction.*

## 2. Materials and Methods

### 2.1. Design

A cross-sectional study was conducted among healthcare professionals working in two public hospitals in Greece (Trikala General Hospital and the Attica Psychiatric Hospital). Data collection took place in clinical departments during the study period. Nurses employed in the participating hospitals were invited to participate using convenience sampling procedures. Inclusion criteria were (i) healthcare professionals employed in the participating hospitals, (ii) age ≥ 18 years, (iii) ability to read and understand Greek, and (iv) provision of informed consent. Exclusion criteria included (i) administrative personnel, (ii) temporary trainees without clinical responsibilities, and (iii) incomplete questionnaires. The sample size was determined based on recommendations for psychometric validation and factor analysis studies. A commonly accepted rule suggests recruiting between 5 and 10 participants per item, with a minimum sample of 200 participants considered adequate for confirmatory factor analysis. Given that the OMIS consists of 20 items, the recommended sample size ranged from 100 to 200 participants. A total of 350 questionnaires were distributed using convenience sampling. The final sample included 301 healthcare professionals yielding a response rate of 86.0%. The total number of participants exceeding these recommendations and providing adequate statistical power for the assessment of the instrument’s psychometric properties.

### 2.2. Ethics

All ethical standards for conducting research were strictly followed. Permission for the use of the study instruments was obtained from the original authors. Ethical approval was granted by the Ethics Committee of the Department of Nursing, University of Thessaly (Protocol No. 16/10 October 2024). Additionally, approval was obtained from the Scientific Councils of both participating hospitals. Data were collected between 1 October and 15 November 2024.

### 2.3. Instruments

The research instrument consisted of two sections. The first section included demographic and occupational characteristics (e.g., gender, age, marital status, education level, years of experience), as well as health-related indicators (e.g., sleep duration, presence of health problems). The second section included the Occupational Moral Injury Scale (OMIS) and the Professional Quality of Life Scale (ProQOL-V).

### 2.4. Occupational Moral Injury Scale (OMIS)

The Occupational Moral Injury Scale (OMIS) was originally developed by Thomas et al. [9] to assess experiences of moral injury in occupational settings. Because no validated Greek version was available, the instrument was translated and culturally adapted following a forward–backward translation procedure and the construct validity and reliability were assessed in present study. The OMIS consists of 21 items, of which 20 contribute to the scoring of five dimensions: Betrayal, Commission with Agency, Commission under Duress, Omission, and Witnessing. Responses are recorded on a 7-point Likert scale ranging from 1 (strongly disagree) to 7 (strongly agree). The Betrayal subscale assesses perceptions of betrayal by leaders, organizations, or trusted authorities. Commission with Agency evaluates distress related to actions that violate one’s moral values and are performed voluntarily. Commission under Duress assesses morally conflicting actions performed under external pressure or coercion. Omission measures distress resulting from failing to act when action was perceived as morally required. Witnessing evaluates exposure to morally troubling events involving the actions of others. The final item is not included in the calculation of subscale scores, as recommended by the original authors.

### 2.5. Professional Quality of Life Scale (ProQOL-V)

The Professional Quality of Life Scale Version 5 (ProQOL-V) [23], previously validated in Greek [24], consists of 30 items distributed across three subscales: Compassion Satisfaction (10 items), Burnout (10 items), and Secondary Traumatic Stress (10 items). Responses are scored on a 5-point Likert scale ranging from 1 (never) to 5 (very often). Compassion Satisfaction measures the positive feelings derived from helping others and performing one’s professional role effectively. Burnout assesses emotional exhaustion, frustration, and feelings of ineffectiveness related to work. Secondary Traumatic Stress evaluates stress symptoms resulting from indirect exposure to other people’s traumatic experiences. Higher scores indicate greater levels of the respective construct. According to established guidelines, scores ≤ 22 indicate low levels, scores between 23 and 41 indicate moderate levels, and scores ≥ 42 indicate high levels.

Data were analyzed using SPSS version 23.0 (IBM Corp., Armonk, NY, USA) and JASP, Version 0.18.3.0 (JASP Team, University of Amsterdam, Amsterdam, The Netherlands) Descriptive statistics included frequencies and percentages for categorical variables, and means, standard deviations, and ranges for continuous variables. Prior to the main analyses, the distribution of continuous variables was assessed for normality using the Shapiro–Wilk test, supplemented by visual inspection of histograms and Q–Q plots. Skewness and kurtosis values were also examined. Variables meeting the assumptions of normality were analyzed using parametric statistical tests.

The factorial validity of the OMIS was evaluated using Confirmatory Factor Analysis (CFA) with the Maximum Likelihood (ML) estimator. Model fit was assessed using multiple goodness-of-fit indices, including the chi-square statistic (χ^2^), Comparative Fit Index (CFI), Tucker–Lewis Index (TLI), Goodness-of-Fit Index (GFI), Standardized Root Mean Square Residual (SRMR), and Root Mean Square Error of Approximation (RMSEA) with its 90% confidence interval. Internal consistency was assessed using Cronbach’s alpha coefficient. Pearson’s correlation coefficient was used to examine relationships between variables. To examine the predictive validity of the OMIS dimensions, three separate multiple linear regression analyses were conducted. The five moral injury dimensions (Betrayal, Commission with Agency, Commission under Duress, Omission, and Witnessing) were entered simultaneously as independent variables, while Compassion Satisfaction (CS), Burnout (BO), and Secondary Traumatic Stress (STS) served as dependent variables. Unstandardized coefficients (B), standardized coefficients (β), 95% confidence intervals, and model fit statistics (F and R^2^) were reported.

## 3. Results

A total of 301 healthcare professionals participated in the study, yielding a response rate of 86%. The majority of the participants were women (72.4%), while men represented 27.6% of the sample. The mean age was 45.8 years (SD = 9.4). Most participants were married (65.1%), followed by single (23.3%) and divorced or widowed (11.6%). Regarding parental status, 38.5% had two children, and 28.6% had none. In terms of education, 39.5% were graduates of Technological Institutes, 29.2% held an MSc degree, and 11% had a university degree. A smaller proportion had a PhD (5.3%) or completed only secondary education (14.6%). Participants were employed in various departments, with the majority working in psychiatric (40.2%) and other departments (20.3%). Similarly, the most common sector was psychiatric (39.9%), followed by laboratory (20.3%) and internal medicine (12.3%). Most participants identified their role as nurse (40.5%), assistant nurse (26.9%) or trainee nurse (15%), while only 8% were head nurses. The majority worked in rotating shifts (63.5%) and held permanent positions (71.4%). The mean working experience was 18.1 years (SD = 10.4). Most participants had a monthly income between €851 and €1450 (83.1%). When asked about their intent to leave their department, 41.5% stated they never intend to leave, while 36.9% said they plan to stay for a few years. Regarding perceptions of the work climate, nearly half (49.7%) rated it as “Good” and 27.3% as “Very Good,” while only a small minority considered it “Bad” or “Very Bad.” (See Table 1 for full details).

### 3.1. Confirmatory Factor Analysis

Since the Occupational Moral Injury Scale (OMIS) had not previously been used in Greece, a standardized forward–backward translation procedure was conducted, followed by a cognitive debriefing process to ensure linguistic and conceptual equivalence of the Greek version.

To examine the factorial structure of the translated instrument, a Confirmatory Factor Analysis (CFA) was performed. Confirmatory factor analysis was conducted using the Maximum Likelihood estimator. The hypothesized five-factor model demonstrated an acceptable-to-moderate fit to the data: χ^2^(160) = 757.32, *p* < 0.001, χ^2^/df ratio = 4.73, CFI = 0.887, TLI = 0.866, IFI = 0.888, GFI = 0.902, SRMR = 0.070, and RMSEA = 0.111 (90% CI = 0.103–0.119). Although the RMSEA exceeded conventional thresholds, other fit indices including SRMR and GFI supported the adequacy of the model. All factor loadings (Table 2) were statistically significant (*p* < 0.001).

### 3.2. Convergent and Discriminant Validity

Convergent validity was examined through the pattern of associations between the OMIS dimensions and theoretically related constructs, including burnout and secondary traumatic stress. As expected, moral injury dimensions were positively correlated with burnout and secondary traumatic stress, supporting convergent validity. In particular, total moral injury was positively associated with burnout (r = 0.384, *p* < 0.001) and secondary traumatic stress (r = 0.344, *p* < 0.001). Discriminant validity was examined through the correlations among the five latent factors. Inter-factor correlations ranged from 0.361 to 0.852. Although some associations were strong, they remained below unity, suggesting that the dimensions are related but not fully redundant. These findings support the multidimensional structure of the scale, while indicating conceptual overlap between some dimensions, particularly Commission with Agency and Commission under Duress (Table 3).

### 3.3. Descriptive Statistics and Reliability

Table 4 presents descriptive statistics and internal consistency indices for all study variables. The Compassion Satisfaction (CS) subscale showed a mean score of 37.30 (SD = 7.35) with good internal consistency (Cronbach’s α = 0.85). Burnout (BO) had a mean of 23.36 (SD = 5.06), while Secondary Traumatic Stress (STS) had a mean of 22.13 (SD = 7.28), both demonstrating acceptable to high reliability (α = 0.79 and α = 0.89, respectively).

Regarding moral injury dimensions, lower mean scores were observed: Betrayal (M = 4.29, SD = 1.37), Commission with Agency (M = 2.70, SD = 1.38), Commission under Duress (M = 2.79, SD = 1.54), Omission (M = 3.32, SD = 1.59), and Witnessing (M = 3.48, SD = 1.65). All subscales demonstrated satisfactory to excellent internal consistency (α = 0.70–0.93). The total OMIS score had a mean of 3.24 (SD = 1.24) and excellent reliability (α = 0.95).

### 3.4. Correlation Analysis

Pearson correlation coefficients (Table 5) revealed that Betrayal was negatively correlated with Compassion Satisfaction (r = −0.151, *p* = 0.009).

In contrast, Betrayal was positively correlated with burnout (r = 0.427, *p* < 0.001) and secondary traumatic stress (r = 0.366, *p* < 0.001).

Commission with Agency was not associated with Compassion Satisfaction (*p* = 0.084), but showed positive correlations with Burnout (r = 0.347, *p* < 0.001) and STS (r = 0.243, *p* < 0.001).

Similarly, Commission under Duress, Omission, and Witnessing were not related to Compassion Satisfaction but were positively and associated with both Burnout and STS (*p* < 0.001).

The total moral injury score was not correlated with Compassion Satisfaction (*p* = 0.610), but was positively associated with Burnout (r = 0.384, *p* < 0.001) and STS (r = 0.344, *p* < 0.001).

### 3.5. Regression Analysis

Three multiple linear regression analyses were conducted to examine the associations between occupational moral injury dimensions and professional quality of life outcomes. Prior to model interpretation, multicollinearity diagnostics were examined. Tolerance values ranged from 0.299 to 0.823 and variance inflation factor (VIF) values ranged from 1.215 to 3.349, indicating acceptable levels of multicollinearity. Condition indices ranged from 1.00 to 12.05, suggesting that collinearity did not materially affect parameter estimation. All regression models were adjusted for demographic and professional characteristics, including age, sex, marital status, years of professional experience, work schedule, job position, and educational level where applicable.

#### 3.5.1. Compassion Satisfaction

The adjusted model was statistically significant (F = 4.16, *p* < 0.001), explaining 10.4% of the variance (Adjusted R^2^ = 0.104). Betrayal (β = −0.202, *p* = 0.001) and Commission with Agency (β = −0.317, *p* = 0.002) were significant negative independent associations with compassion satisfaction, whereas Commission under Duress was positively associated with compassion satisfaction (β = 0.221, *p* = 0.031). Omission and Witnessing were not significant predictors.

#### 3.5.2. Burnout

The adjusted model was statistically significant (F = 8.16, *p* < 0.001), explaining 22.3% of the variance (Adjusted R^2^ = 0.223). Betrayal (β = 0.357, *p* < 0.001) and Commission with Agency (β = 0.262, *p* = 0.006) were significant positive independent associations with burnout. No significant associations were observed for Commission under Duress, Omission, or Witnessing.

#### 3.5.3. Secondary Traumatic Stress

The adjusted model was statistically significant (F = 8.65, *p* < 0.001), explaining 23.5% of the variance (Adjusted R^2^ = 0.235). Betrayal (β = 0.253, *p* < 0.001) was positively associated with secondary traumatic stress. None of the remaining occupational moral injury dimensions were independently associated with secondary traumatic stress after adjustment for covariates, although Omission approached statistical significance (β = 0.144, *p* = 0.099).

Overall, Betrayal emerged as the most consistent correlate of poorer professional quality of life, being associated with lower compassion satisfaction and higher levels of burnout and secondary traumatic stress. Commission with Agency was associated with lower compassion satisfaction and higher burnout, whereas the associations of the remaining occupational moral injury dimensions were attenuated after adjustment for demographic and professional characteristics. Detailed results are presented in Table 6.

## 4. Discussion

The present study provides evidence supporting the reliability and factorial validity of the Greek version of the Occupational Moral Injury Scale (OMIS), contributing to the limited literature on moral injury within the Greek healthcare context. The findings indicate that occupational moral injury is significantly associated with key dimensions of professional quality of life, particularly burnout and secondary traumatic stress. These results are consistent with prior research suggesting that moral injury constitutes a significant psychosocial risk factor for healthcare professionals [6,12,13].

Although the RMSEA value (0.111) exceeded the conventionally recommended threshold, recent methodological literature has cautioned against evaluating CFA models based on RMSEA alone. Kenny et al. (2015) [25] noted that RMSEA may sometimes overestimate model misfit and should be interpreted in conjunction with other fit indices rather than as a sole criterion of model adequacy. Similarly, Shi et al. (2022) [26] emphasized that fit evaluation should rely on a combination of indices, as the performance of individual fit statistics may vary depending on model characteristics and sample conditions. Therefore, model fit in the present study was assessed using multiple complementary indices. The observed values for SRMR (0.070), GFI (0.902), CFI (0.887), and IFI (0.888), together with statistically significant factor loadings and theoretically meaningful factor correlations, provide evidence supporting an acceptable-to-moderate fit of the proposed five-factor model. Nevertheless, the elevated RMSEA suggests that the model fit is not optimal and should be interpreted with appropriate caution [25,26]. Beyond statistical considerations, the moderate fit indices observed in the present study may also reflect cultural and contextual differences in how moral injury is experienced and interpreted within healthcare systems. The OMIS was originally developed and validated in English-speaking populations, and some dimensions of moral injury may manifest differently in nurses working within the Greek public healthcare sector.

Among the dimensions examined, *Betrayal* emerged as the most consistent and robust independent associations of adverse outcomes. It was positively associated with both burnout and secondary traumatic stress, while negatively associated with compassion satisfaction. This finding highlights the critical role of perceived organizational and interpersonal trust. Experiences of betrayal—whether related to leadership, institutional constraints, or ethical conflicts—have been shown to significantly undermine psychological well-being and professional functioning [12,13,27]. Similar findings have been reported in recent meta-analyses, demonstrating moderate to strong associations between moral injury and mental health outcomes such as burnout, anxiety, and depression [28].

In contrast, *Commission with Agency* demonstrated a dual effect, being negatively associated with compassion satisfaction and positively associated with burnout. This suggests that direct involvement in morally distressing actions, particularly when accompanied by a sense of personal responsibility, intensifies emotional burden and reduces the positive aspects of caregiving. This observation aligns with previous findings indicating that personal accountability in morally conflicting situations is a core component of moral injury [9,29]. Similarly, *Omission* was specifically associated with secondary traumatic stress, supporting the notion that failure to act in ethically challenging situations may lead to trauma-related symptoms [30]. In contrast, *Witnessing* did not demonstrate significant associations with ProQOL-V dimensions, highlighting the distinction between passive exposure and active moral involvement, as suggested in the original conceptualization of OMIS [9].

The descriptive findings revealed high levels of compassion satisfaction alongside moderate levels of burnout and secondary traumatic stress. This pattern supports the growing body of literature indicating that positive and negative dimensions of professional quality of life may coexist [1,31]. Compassion satisfaction appears to function as a protective factor, mitigating the psychological impact of occupational stress. Previous research has demonstrated that deriving meaning and fulfillment from caregiving roles can buffer against emotional exhaustion and secondary trauma [32].

The protective role of compassion satisfaction may be further enhanced by factors such as professional experience, job stability, and supportive work environments. In addition, self-compassion has been identified as a significant moderator of professional quality of life. For instance, Mangoulia et al. [33] found that higher levels of self-compassion were associated with lower burnout and secondary traumatic stress and higher compassion satisfaction among healthcare professionals in Greece. These findings underscore the importance of fostering supportive organizational cultures that promote emotional regulation, reflective practice, and resilience.

An unexpected finding was the positive association between Commission under Duress and Compassion Satisfaction in the multivariable model despite the absence of a significant bivariate relationship. This pattern may reflect a statistical suppression effect resulting from overlap among the dimensions of moral injury. Consequently, this finding should be interpreted cautiously and requires replication in future studies.

The observed levels of moral injury suggest that healthcare professionals encounter ethically challenging situations in their clinical practice. However, because prevalence estimates were not calculated, the findings should not be interpreted as indicating the prevalence of moral injury within the healthcare workforce. This is particularly relevant in healthcare systems characterized by high workload, limited resources, and complex ethical demands, such as the Greek public sector [10,29]. Previous studies conducted during the COVID-19 pandemic further highlighted how such conditions exacerbate moral distress and contribute to long-term psychological consequences [14,15].

Although moral injury explained significant proportions of burnout and secondary traumatic stress, the modest R^2^ values indicate that professional quality of life is influenced by multiple organizational, interpersonal, and individual factors beyond moral injury. Contemporary occupational health models emphasize that burnout and work-related psychological distress arise from complex interactions among individual characteristics, organizational culture, workload, staffing adequacy, leadership quality, social support, and resilience-related resources. Therefore, while moral injury contributes meaningfully to professional well-being, a substantial proportion of variance remains attributable to factors not assessed in the present study.

One of the most noteworthy findings of the present study was the consistent association between the betrayal dimension of moral injury and both burnout and secondary traumatic stress. This finding suggests that the psychological impact of moral injury may extend beyond exposure to ethically challenging situations themselves and may be particularly influenced by how healthcare professionals perceive the actions of the organizations, leaders, and systems within which they work. When individuals feel unsupported, unheard, or abandoned by those they trust, the resulting sense of betrayal may undermine professional commitment, emotional well-being, and confidence in the healthcare environment.

This interpretation is consistent with research on ethical climate [34], which emphasizes the importance of organizational fairness, open communication, and supportive leadership in promoting staff well-being. Previous research among Greek nurses has demonstrated that perceptions of a positive hospital ethical climate are associated with better work-related quality of life and more favorable professional outcomes [35]. Similarly, Greek nurses who perceived their work environment as ethically supportive reported more positive evaluations of their organizational climate [36]. Nurses who perceive their workplace as ethically supportive are generally better equipped to cope with workplace stressors, whereas environments characterized by inadequate support or perceived injustice may increase vulnerability to emotional exhaustion and psychological distress. Similarly, the concept of moral distress suggests that repeated exposure to situations in which professionals feel unable to act according to their ethical values, particularly when organizational constraints are involved, may contribute to feelings of frustration, helplessness, and burnout [37,38]. Viewed through these perspectives, betrayal may reflect not only an individual experience but also broader organizational and relational shortcomings that shape how healthcare professionals experience and respond to morally challenging situations.

From a clinical and organizational perspective, these findings highlight the importance of creating supportive ethical work environments. Potential organizational strategies include ethics consultation services, structured clinical supervision, peer-support programs, reflective practice groups, leadership training focused on ethical decision-making, and interventions designed to strengthen trust between healthcare professionals and management [20]. Such initiatives may help mitigate the psychological burden associated with morally challenging clinical situations.

### Strengths and Limitations

This study has several strengths. It represents the first psychometric evaluation of the Greek version of the OMIS among healthcare professionals and includes a relatively large sample from two public hospitals. In addition, the use of validated instruments and multivariable analyses enabled a comprehensive examination of the associations between occupational moral injury and professional quality of life.

Several limitations should also be acknowledged. First, the cross-sectional design does not permit conclusions regarding causality or temporal relationships among variables. Second, all measures relied on self-report questionnaires, which may be subject to recall bias, social desirability bias, and common-method variance. Third, participants were recruited from only two public hospitals using a convenience sampling approach, limiting the generalizability of the findings. Fourth, although the CFA provided support for the proposed factorial structure of the OMIS, some fit indices indicated only marginal-to-moderate model fit and therefore warrant cautious interpretation. Fifth, the correlation between Commission with Agency and Commission under Duress was high (r = 0.852), suggesting substantial shared variance between these dimensions. Although the original five-factor structure was retained in accordance with the theoretical framework of the OMIS, future studies should further investigate discriminant validity using HTMT ratios and Fornell–Larcker criteria. Finally, the regression analyses were based on composite scores derived from 5-point and 7-point Likert scales. Although this approach is widely accepted in psychometric research, such scales provide limited response categories and may not capture subtle variations as effectively as continuous measures. Future studies could consider instruments with a broader response range or continuous variables to improve measurement precision and potentially increase the explanatory power of the regression models.

## 5. Conclusions

This study contributes to the emerging literature on occupational moral injury among healthcare professionals by providing the first psychometric evaluation of the Greek version of the OMIS. The findings indicate that higher levels of occupational moral injury, particularly betrayal and commission with agency, were associated with higher levels of burnout and secondary traumatic stress and lower levels of compassion satisfaction.

Given the cross-sectional design, these findings should be interpreted as associations rather than causal relationships. Nevertheless, the results underscore the importance of organizational initiatives that promote ethical support, leadership trust, reflective practice, and clinical supervision. Healthcare institutions should consider implementing ethics-support frameworks, resilience-building interventions, and mechanisms that facilitate transparent communication and shared decision-making.

Future multicenter and longitudinal studies are needed to further evaluate the psychometric properties of the Greek OMIS and to clarify the temporal relationships between occupational moral injury and professional quality-of-life outcomes.

## Figures and Tables

**Table 1:** Demographic and Work-related Characteristics.

Variable	Level	Counts	Proportion (%)
Sex	Male	83	27.6
	Female	218	72.4
Age Mean (SD)			45.8 (9.4)
Marital Status	Single	70	23.3
	Married	196	65.1
	Divorced/Widowed	35	11.6
Number of Children	0	86	28.6
	1	52	17.3
	2	116	38.5
	3	35	11.6
	4	12	4.0
Educational Status	University	33	11.0
	Technological Institute	119	39.5
	MSc	88	29.2
	PhD	16	5.3
	Secondary Education	45	15
Hospital type	General	181	60.1
	Psychiatric	120	39.9
Job Department	ICU	10	3.3
	Internal Medicine	27	9.0
	Emergency	34	11.3
	Technological	32	10.6
	Surgical	6	2.0
	Cardiology	10	3.3
	Psychiatric	121	40.2
	Other ^1^	61	20.3
Position	Head Nurse	24	8.0
	Supervisor	29	9.6
	Nurse	122	40.5
	Assistant Nurse	81	26.9
	Other ^2^	45	15.0
Shift Type	Morning	110	36.5
	Rotating	191	63.5
Employment Status	Permanent	216	71.8
	Contract	85	28.2
Working Experience Mean (SD)			18.1 (10.4)
Monthly Income (€)	≤850	20	6.6
	851–1450	250	83.1
	≥1451	31	10.3
Intent to Leave Department	Immediately	17	5.6
	Soon	20	6.6
	Within 1 Year	28	9.3
	Stay for a Few Years	111	36.9
	Never Leave	125	41.5
Work Climate	Very Good	83	27.3
	Good	149	49.7
	Neutral	57	19.0
	Bad	7	2.3
	Very Bad	5	1.7

^1^. Outpatient clincs. ^2^. Nurse in specialty training.

**Table 2:** Unstandardized Regression Weights (B) and Standardized Factor Loadings (λ) for the Five-Factor Model of the Occupational Moral Injury Scale.

Factor	Item	Unstandardized Regression Weights (B)	Standardized Loading (λ)	*p*
Betrayal	1	1.252	0.78	<0.001
	2	1.394	0.85	<0.001
	3	1.044	0.62	<0.001
Commission with Agency	4	1.133	0.72	<0.001
	5	1.240	0.85	<0.001
	6	1.441	0.85	<0.001
	7	1.390	0.85	<0.001
	8	1.393	0.85	<0.001
Commission under Duress	9	1.393	0.79	<0.001
	10	1.588	0.86	<0.001
	11	1.559	0.88	<0.001
	12	1.407	0.82	<0.001
Omission	13	1.721	0.83	<0.001
	14	1.644	0.80	<0.001
	15	1.662	0.84	<0.001
	16	1.092	0.57	<0.001
Witnessing	17	1.409	0.76	<0.001
	18	1.570	0.84	<0.001
	19	1.705	0.84	<0.001
	20	1.550	0.74	<0.001

Note. B = unstandardized regression weight; λ = standardized factor loading. All factor loadings were statistically significant at *p* < 0.001. Standardized factor loadings ranged from 0.57 to 0.88.

**Table 3:** Inter-Factor Correlations among the Dimensions of the Occupational Moral Injury Scale–Health Professional Version.

Factor	1	2	3	4	5
1. Betrayal	1.00				
2. Commission with Agency	0.378 **	1.00			
3. Commission under Duress	0.361 **	0.852 **	1.00		
4. Omission	0.397 **	0.702 **	0.728 **	1.00	
5. Witnessing	0.377 **	0.686 **	0.622 **	0.741 **	1.00

** *p* < 0.001.

**Table 4:** Descriptive Statistics for the Scales Used in the Study.

Scale	Mean	Standard Deviation	Minimum	Maximum	Cronbach’s α
Compassion Satisfaction (CS)	37.30	7.35	10.00	50.00	0.85
Burnout (BO)	23.36	5.06	12.00	40.00	0.79
Secondary Traumatic Stress (STS)	22.13	7.28	10.00	46.00	0.89
Betrayal	4.29	1.37	1.00	7.00	0.70
Commission with Agency	2.70	1.38	1.00	6.60	0.91
Commission under Duress	2.79	1.54	1.00	7.00	0.93
Omission	3.32	1.59	1.00	7.00	0.85
Witnessing	3.48	1.65	1.00	7.00	0.90
Total Occupational Moral Injury	3.24	1.24	1.00	6.45	0.95

**Table 5:** Pearson’s Correlations between ProQol-V and Occupational Moral injury.

Variable		CS	BO	STS
Betrayal	Pearson’s r	−0.151	0.427	0.366
	*p*-value	0.009	<0.001	<0.001
Commission with Agency	Pearson’s r	−0.100	0.347	0.243
	*p*-value	0.084	<0.001	<0.001
Commission under Duress	Pearson’s r	0.023	0.260	0.299
	*p*-value	0.697	<0.001	<0.001
Omission	Pearson’s r	0.043	0.283	0.309
	*p*-value	0.457	<0.001	<0.001
Witnessing	Pearson’s r	0.031	0.285	0.234
	*p*-value	0.593	<0.001	<0.001
Total Occupational Moral Injury	Pearson’s r	−0.030	0.384	0.344
	*p*-value	0.610	<0.001	<0.001

**Table 6:** Adjusted Multiple Linear Regression Analyses Predicting Professional Quality of Life Outcomes from Occupational Moral Injury Dimensions.

**Compassion Satisfaction (CS)**						
**Predictor**	**B**	**SE**	**β**	**95% CI** **Lower Upper**		***p***
Betrayal	−1.071	0.328	−0.202	−1.718	−0.424	0.001
Commission with Agency	−1.671	0.534	−0.317	−2.723	−0.619	0.002
Commission under Duress	1.047	0.483	0.221	0.097	1.997	0.031
Omission	0.344	0.427	0.075	−0.496	1.185	0.421
Witnessing	0.418	0.366	0.094	−0.303	1.139	0.255
**Model statistics: F(11,288) = 4.16, *p* < 0.001, Adjusted R^2^ = 0.104.**						
**Burnout (BO)**						
**Predictor**	**B**	**SE**	**β**	**95% CI** **Lower Upper**		***p***
Betrayal	1.310	0.211	0.357	0.894	1.726	<0.001
Commission with Agency	0.953	0.344	0.262	0.277	1.630	0.006
Commission under Duress	−0.444	0.317	−0.136	−1.068	0.180	0.162
Omission	0.229	0.277	0.072	−0.317	0.774	0.410
Witnessing	0.110	0.237	0.036	−0.356	0.576	0.642
**Model statistics: F(12,287) = 8.16, *p* < 0.001, Adjusted R^2^ = 0.223.**						
**Secondary Traumatic Stress (STS)**						
**Predictor**	**B**	**SE**	**β**	**95% CI** **Lower Upper**		***p***
Betrayal	1.340	0.303	0.253	0.743	1.937	<0.001
Commission with Agency	−0.087	0.494	−0.016	−1.058	0.885	0.861
Commission under Duress	0.571	0.455	0.121	−0.325	1.468	0.211
Omission	0.658	0.398	0.144	−0.126	1.442	0.099
Witnessing	−0.132	0.340	−0.030	−0.801	0.537	0.697
**Model statistics: F(12,287) = 8.65, ***p*** < 0.001, Adjusted R^2^ = 0.235.**						

Note. Regression models were adjusted for demographic and professional characteristics, including age, sex, marital status, years of professional experience, educational level, work schedule, and job position. Only coefficients for the Occupational Moral Injury Scale dimensions are presented.

## Data Availability

Data are available from the corresponding author upon request. The data are not publicly available due to privacy or ethical restrictions.
